# 

**Published:** 1838-04-01

**Authors:** 


### BIBLIOGRAPHICAL RECORD.

1. The Naturalist; illustrative of the Animal, Vegetable, and Mineral Kingdoms—with engravings. In monthly numbers, price 2s. No. 16, January 1, 1838. Whittaker and Co.

☞ *This is a very amusing and instructive periodical.*

2. Report of the Proceedings under a Brieve of Idiotcy—Peter Duncan *v.* David Yoolow, tried at a Cupar-Angus in January 1837. With an Appendix, &c. By Ludovic Colquhoun, Esq. Advocate. Octavo, pp. 116. Clark, Edin. 1838.

3. The Quarterly Journal of the Calcutta Medical and Physical Society. Edited by Drs. Goodeve & O'Shaughnessey. Nos. 1 and 2, January and April, 1837.

☞ *This Journal bids fair to advance the interests of medical science in the East, by diffusing knowledge more extensively and cheaply than could be done through the medium of British periodicals transmitted to India.*

4. Transactions of the Medical and Physical Society of Calcutta. Vol. VII. Part 1. Calcutta, 1836.

5. Syllabus of Lectures on Materia Medica and Therapeutics; containing a Physiological Arrangement of the Materia Medica. By O'Bryen Bellingham, M. D. Surgeon to the St. Vincent's Hospital, Dublin, &c. &c. Hodges and Smith, 1837.

☞ *The arrangement is ingenious, and chiefly selected from the arrangements employed or suggested by others, as Murray, Granville, &c. but probably improved.*

6. A Letter to the Inhabitants of Ceylon on the Advantages of Vaccination. By J. Kinnis, M.D.

7. Reports and other Documents relating to the State Lunatic Hospital, at Worcester, Mass. Printed by order of the Senate. Octavo. Boston, 1837.

8. The Works of John Hunter, F. R. S. with Notes. Edited by Jas. F. Palmer, Esq. &c. In four volumes—Vol. IV. illustrated by a Volume of Plates in 4to. 1838.

9. Pharmacopœia Collegii Regalis Medicorum Londinensis. London—Woodfall, 1836.—*A very neat pocket-book.*

---

10. Memoirs on the Nervous System. By Marshall Hall, M.D. &c. &c. 4to. With Plates. Sherwood & Co. 1837.

---

11. Lectures on the Nervous System. By Marshall Hall, M.D. 8vo. 1836.

---

12. An Essay on the Atiquity of Hindoo Medicine, including an introductory Lecture on the Course of Materia Medica and Therapeutics delivered at King's College. By J. F. Royale, M.D. F.R.S. &c. Octavo, pp. 196. Allen, Leadenhall Street, 1838.

☞ *This is a work of immense research and erudition. It will prove a source of great interest amongst our medical brethren, and indeed most Europeans of education, in India.*

---

13. Ophthalmia. The various Inflammations of the Conjunctiva, or Mucous Membrane of the Eye. By J. Slade, M.D. &c. lately Physician to the North Devon Hospital, &c. Octavo, pp. 120. Parbury and Co. Leadenhall Street, 1838.

---

14. Elements of Anatomy. By Jones Quain, M.D. Fourth edition, Part 2,—Price 5s. Taylor and Walton, 1838.

---

15. Elements of Chemistry, including the recent Discoveries, &c. &c. By the late Edward Turner, M. D. Sixth Edition, enlarged and revised, by Justus Liebig and Wilton G. Turner. Part 2, price 5s. Taylor and Walton, 1838.

---

16. A Lecture introductory to the Business of the Original School of Medicine, Peter Street, Dublin. By G. T. Hayden, A.B. M.R.C.S.I. Lecturer on Anatomy, &c.

☞ *We have made an extract from this humorous Address in another part of the Journal.*

---

17. Appendix to the Eighth Edition of the Pharmacologia; with some Remarks on various Criticisms upon the London Pharmacopœia of 1836. By J. A Paris, M.D. Octavo, pp. 31. Highley, 32, Fleet Street, January 1838—price 2s. 6d.

---

18. Medicine and Surgery one inductive Science; being and Attempt to improve its Study and Practice on a plan in closer alliance with inductive Philosophy, and offering, as first fruits, the Law of Inflammation; addressed particularly to the Medical Student and the Profession; but easy and intelligible to the Public also; the whole being the introduction and first part of a System of Surgery. By Geo. Macilwain, Surgeon to the Finsbury Dispensary, &c. Octavo, pp. 550. Highley, Jan. 1838.

---

19. The Cyclopædia of Anatomy and Physiology. Edited by R. B. Todd, M.D. &c. Part XII. containing Gasteropoda (T. R. Jones, Esq.)—Gelatin (W. T. Brande)—Generation, Organs of (T. Rymer)—Generation (Dr. A. Thomson)—Gland (R. D. Grainger)—illustrated with numerous engravings. Sherwood and Co. 1838.

---

20. Elements of Physiology. By J. Muller, M.D. Translated from the German, with Notes, by William Baly, M.D. Illustrated with steel plates, &c. Part 2, containing Secretion, Digestion, Function of the Glands without Efferent Ducts, and Excretion. London—Taylor and Walton, Feb. 1838. Price 3s. 6d.

---

21. The Chirurgico-Comico-Hieroglifico Dictionary. By Pill-Box. Nos. 2 and 3, price 1s. each, containing 6 amusing Etchings under their respective letters.

---

22. The Student's Companion to Apothecaries' Hall; or the London Pharmacopœia of 1836, in question and answer. By Ed. Oliver, M.R.C.S.L. Duodecimo. Churchill, Feb. 1838.

☞ *This is not a string of solutions to catch questions, but a scientific delineation and exposition of the Pharmacopœia in a colloquial form, calculated to remain more tenaciously in the mind of the Student, than through the medium of dry details.*

---

23. A short Treatise on the external Characters, Nature, and Treatment of the different Forms of Porrigo, Scalled-head, and Ringworm. By Walter Dick, M.D. &c. formerly House-Surgeon to the Glasgow Royal Infirmary. Pp. 58. Glasgow, Feb. 1838.

---

24. Traité Theorique et Pratique de la Derivation, contre les Affections les plus communes en général, tel que la Plethore, l'Inflammation, &c. Par L. F. Gondret, M.D. &c. Paris, 1837.

☞ *Some notice of this little Work will be found in the Review department.*

---

25. A brief Account of the Application and Uses of the Utero-abdominal Supporter—a new Instrument for the Relief and Cure of Procidentia and Prolapsus Uteri.

Patented by A. G. Hull, M.D. &c. Higham, Agent, 52, Regent Street.

---

26. Elements of Anatomy. By Jones Quain, M.D. Part III. price 5s. completing the Work. Fourth edition, revised and corrected. Taylor and Co. Feb. 1838.

---

27. Series of Anatomical Plates. Fas. 56, Division III.—Nerves. By Dr. Quain. Price 21s. Feb. 1838.

---

28. The Philosophy of Marriage, in its Social, Moral, and Physical Relations; with an Account of the Diseases of the Genito-Urinary Organs, which impair or destroy the re-productive Function, and induce a variety of Complaints; with the Physiology of Generation in the Vegetable and Animal Kingdoms, &c. By Michael Ryan, M.D. &c. Churchill, 1838.

---

29. Notes on the Medical Topography of Calcutta. By Jas. Ranald Martin, Esq. Presidency Surgeon, and Surgeon to the Native Hospital. Octavo, pp. 181. Calcutta, 1837.

---

30. A Treatise on Ruptures. By William Lawrence, Esq. F.R.S. Fifth Edition, revised, corrected, and considerably enlarged. Churchill, London, 1838.

---

31. A practical Treatise on the Curative Effects of simple and medicated Vapour applied locally in Rheumatism, Gout, White Swelling, and other Diseases of the Joints, &c. &c. With engravings of the Apparatus. By Jas. Wilson, M.D. M.R.C.S. Octavo, pp. 145. Churchill, 1838.

---

32. The Continental and British Medical Review, &c. By A. M. Bureaud Riofrey, M.D. January, February, and March, 1838. *In exchange.*

---

33. The Phrenological Journal, and Magazine of Moral Science—No. 2, March 1838. *In exchange.*

---

34. The Dublin Journal of Medical Science, &c. March, 1838. *In exchange.*

---

35. The India Review; and Journal of Foreign Science and the Arts. For March, April, and July, 1837. Edited by F. Corbyn, Esq.. *In exchange.*

---

36. The India Journal of Medical and Physical Science. By F. Corbyn, Esq. For March, April, May, and August, 1837. *In exchange.*

37. Ophthalmic Memoranda respecting those Diseases of the Eye, which are more frequently met with in practice. By John Foote, F.R.C.S. &c. Renshaw, Strand, 1838. Price 1s.

☞ *This little Vade-mecum would go almost into a thimble, and contains a great deal of concentrated information.*

---

38. On the Functions of the Cerebellum. By Drs. Gall, Vimont, and Broussais. Translated from the French by George Combe—also, Answers to the Objections urged against Phrenology by Drs. Roget, Rudolphi, Richard, and Tiedemann. By George Combe & Dr. Coombe. Octavo, Machlachlan & Stewart, Edin. 1838.

---

39. Experimental Inquiry into the Physiology of Cutaneous Absorption, and its Application to Therapeutics, &c. By William Herries Madden, M.D. &c. Octavo, John Caffrae & Son, Edin. 1838.

---

40. A History of British Birds. By W. Yarrell, F.L.S. &c. Part 5, price 2s. 6d.

---

41. A History of British Reptiles. By Thos. Bell, F.R.S. Part 1, price 2s. 6d. illustrated by a Wood-cut of each Species, &c. London—John Van Voorst, March, 1838.

---

42. A Treatise on the Nature, Symptoms, Causes, and Treatment of Insanity; with practical Observations on Lunatic Asylums, &c. By Sir W. C. Ellis, M.D. resident Medical Superintendant of the Wakefield Asylum. Octavo. Holdsworth, London, March 1838.

---

43. The British Flora Medica; or History of the Plants of Great Britain. Illustrated by a coloured figure of each plant. By B. H. Barton, F.L.S. & Thos. Castle, M.D. F.L.S. Vol. the 2nd and last, with an engraving of Linnæus. Cox, St. Thomas-st. Borough, 1838.

---

44. A Treatise on some Nervous Affections; being chiefly intended to illustrate those Varieties which simulate Structural Disease. By Ed. Lee, M.R.C.S. Second Edition, re-written and considerably enlarged. Churchill, London, March 1838.

---

45. A Botanical Lexicon; or Expositor of the Terms, Facts, and Doctrines of the Vegetable Physiology, brought down to the present time. By the Rev. Patrick Keith, F.L.S. &c. Octavo, pp. 416. Wm S. Orr and Co. 1838.——*In our next.*

### INDEX.

A

Abdominal pulsation, Dr. Johnson on, 569
Absorbents of penis, inflammation of, 330
Absorption, Magendie on .......... 527
Acalepha .................... 421
Accidents from swallowing acids .... 399
Acids, accidents from swallowing.... 399
Acrid diuretics ................... 110
Action of diuretic medicines, Dr. Mitcherlich on the ................. 107
Acupuncture for hydrocele ......... 563
Acute primary bronchitis .......... 37
Acute secondary bronchitis ........ 44
Acute phthisis, illustrations of ...... 270
Addison on electricity in convulsive and spasmodic diseases .......... 517
Administration of mercury ........ 67
Albuminaria, cases illustrative of.... 542
Alkaline diuretics ................ 111
Alleged affection of organs of calculation 243
Amaurosis and cataract, diagnosis of.. 559
Amaurosis, treatment of, by cauterization of the cornea .............. 560
Amphibia, nervous system of the.... 412
Amphibia, brain of the .... ...... 414
Anatomical plates, by Quain........ 116
Anatomy of the spinal cord...... 123, 361
Anatomy of the spinal nerves ...... 124
Anatomy of sub-maxillary ganglion.. 366
Anatomy, Dr. Quain's elements of .. 469
Animalculæ, spermatic, experiments on 529
Aneurysm, cases of, by Mr. Cooper.. 215
Aneurysm of the iliac artery, spontaneous cure of an .............. 567
Anus, imperforation of the ........ 4
Anus, inflammation and excoriation of 20
Anus, fissure of the .............. 20
Anus, tumors at the verge of the.. . 307
Aphorisms of Hippocrates, commentaries on the .................... 276
Arachnoid, lesions of the .......... 451
Arachnoid, external to the brain .... 451
Arm, injury of the, from musket-shot, 291
Arm, fractures of the ............. 585
Arsenicated candles .............. 459
Articulata, digestive organs of the.... 421
Ashwell on the use of the speculum.. 519
Asphyxia, bronchotomy in ........ 410
Ausculation, M. Louis on .......... 257
Auscultatory phenomena, Magendie on 521

B.

Bienaymé on duration of life in France 534
Bilious remittent fever ............ 490
Bilious remittent fever, causes of .... 491
Bilious remittent fever, treatment of, 495
Blake on monomania. ............. 84
Blakiston on the influenza.......... 168
Brachet on rheumatism ........... 538
Brain in the negro, organization of the 249
Brain in the negro, weight of the .... 251
Brain in the European, weight of the, 249
Breach of etiquette ............... 617
Breast, tumors of the.............. 142
Breast, irritable tumors of the ...... 144
Breast, cancer of the, in the male.... 148
Bricheteau's clinical lectures........ 264
Bricheteau on phthisis pulmonalis .. 264
British Legion, med. history of...... 387
Bronchial respiration .............. 259
Bronchitis ........................ 35
Bronchitis, physical signs of ........ 41
Bronchitis of typhus ..... ......... 44
Bronchitis, treatment of. ........... 47
Bronchitis, treatment of second stage, 47
Bronchotomy for asphyxia.......... 410
Broussais on education ............ 238
Bubo .......................... 329
Bubo, circumstances under which it occurs ............... ...... 329
Bubo, properties of the discharge from 340
Bubo, treatment of................. 341
Bubo, treatment of, in the stage of suppuration .................... 347
Bubo, obstinate ulcerations from .... 355
Burke on fract. of lower extremities, 171
Burns, treat. of with diachylon plaster 562
Bushe on diseases of the rectum .... 1
Bushe on the rectum and anus...... 297
Bye-laws of the Royal College of Surgeons in Ireland .......... ...... 622

C.

Cachexia aquosa in Egypt .......... 553
Calcareous degeneration of the larynx, 394
Camphor in rheumatism ........... 554
Cancer of the breast, in the male.... 148
Capsular rheumatism............ .. 224
Carbonic acid for dysmenorrhœa .... 554
Cases of fracture .................. 551
Causes of hæmorrhoidal tumors .... 309
Cephalalgia, intense, case of ........ 553
Charactères phrenologiques ......... 227
Character of Cuvier .............. 236
Cheltenham Dispensary, Dr. Dickson, 621
Chemistry, elements of, by Dr. Turner 174
Children, remittent fever of ........ 481
Chlorotic fever .................... 192
Cholera, treatment of .. .......... 164
Chronic primary bronchitis ........ 38
Chronic secondary bronchitis ...... 45
Classification of the nervous system.. 371
Climate, influence of, on phthisis pul. 267
Clinical lectures, by M. Bricheteau .. 264
Clinical lectures, by Mr. Guthrie, 289, 585
Clutterbuck on pyrexia............ 153

Cock on the ganglionic enlargement of the pneumo-gastric nerve ........ 361
Colles on the venereal disease .... 65, 329
Comparative anatomy, Dr. Grant on, 412
Comparative anatomy of digest. organs 417
Complete laceration of the rectum .. 14
Compound fracture of the elbow .... 281
Congestion of hæmorrhoidal vessels.. 297
Contagion, Magendie on ........... 522
Contagiousness of typhus .......... 523
Contagiousness of cholera.......... 524
Convulsive and spasmodic diseases, electricity in .................. 517
Cooper on aneurysm ............... 215
County of Clare Infirmary, report of, 281
Cranium, capacity of the cavity of the 251
Creosoton ........................ 467
Crotchet case ..................... 511
Croup ............................ 53
Croup, treatment of .............. 55
Croup, mercurial treatment of ...... 58
Cuvier, character of .............. 236
Cyclo-neurose, digestive organs of the, 418
Cyclopædia of anatomy and physiology 378

D.

Delirium, case of.................. 244
Demonomania with delirium......... 245
Derivation, Dr. Gondret on ........ 457
D'Espine on phthisis ............... 270
Diagnosis of thoracic disease........ 34
Diagnosis of nervous palpitation .... 436
Diagnosis of amaurosis and cataract.. 555
Diagnostic characters of inflammation, 447
Dickson, Dr. and Dr. Johnson ...... 285
Dickson, Dr. & Cheltenham Dispensary 621
Disksonianism at home and abroad ... 578
Digestive organs, comparative anat. of 417
Digestive organs of the cyclo-neurose, 418
Digestive organs of the articulata.... 421
Digestive organs of the higher animals, 424
Digestive tubes, lesions of the ...... 547
Digitalis, action of, on the heart .... 113
Diglena lacustris, digestive organs of, 422
Dipthérite ......................... 51
Discharge from bubo, properties of the 340
Disease of the kidneys ............. 542
Diseased veins, Dr. Monat on ....... 574
Diseases of the rectum ............ 1
Diseases of the rectum, Mr. Syme on, 1
Diseases of the chest and windpipe .. 33
Diseases of the chest, Dr. Stokes on.. 33
Diuretic medicines, Dr. Mitcherlich on the action of .................. 107
Diuretics, saline and alkaline......... 111
Diuretics, action of, on the kidneys .. 111
Diuretics, causing an increased secretion of urine .................. 113
Division of the tendo-achillis........ 221
Dropsy.......................... 114
Dumfries & Galloway Infirmary report 205
Duration of life in France .......... 534
Dysmenorrhœa, carbonic acid for.... 554

E.

Echinoderma, digestive organs of the, 421
Education, M. Broussais on ........ 238
Education, application of phrenology to 238
Egypt, cachexia aquosa in........... 553
Elbow, compound fracture of the .... 281
Elbow-joint, excision of the ........ 613
Elements of anatomy, by Quain, 116, 469
Elements of physiology, Müller's, 116, 469
Encephalic irritation, M. Piorry on .. 252
Entozoa, digestive organs of the .... 421
Epidemic diseases, remarks on ...... 554
Epidemic, causes of ............... 540
Epigastric pulsations, causes of...... 571
Etiquette, breach of .............. 617
Evils of the factory system in France, 535
Excision of the head of the femur.... 608
Excision of the elbow-joint ........ 613
Excito-motory actions, seat of the.... 363
Experiments on spermatic animalculæ, 529
External hæmorrhoids ............ 309
External hæmorrhoids, treatment of 323
Extirpation of the uterus .......... 561
Extremity of the rectum, neuralgia of, 24
Eye, Dr. Walker on the ............ 491
Eyelids, encysted tumors of the...... 515

F.

Factory system in France, evils of the 535
Farr's British Medical Almanack .... 175
Fatal hæmorr. from wound of tongue, 217
Fatal injury of the leg, case of ...... 282
Fatalism, opposition of phrenology to, 241
Femur, spontaneous fracture of the.. 551
Femur, excision of the head of the .. 608
Fever from prolonged lactation.. 205, 473
Fever, remittent, of children......... 481
Fever, bilious remittent............ 490
Fevers, Dr. M'Divitt on............ 186
Fissure of the anus................ 21
Flora of Jamaica ................. 170
Florens on phrenology ............ 241
Fluid products of inflamed tissues.... 449
Fœtus heard to cry before death .... 531
Forceps case ...................... 511
Forearm, peculiar affection of the.... 549
Foreign bodies in the larynx & trachea 402
Forget on disease of the kidneys .... 542
Fracture of patella by muscular effort, 551
Fracture of the femur, spontaneous, 551
Fractures, cases of ............... 551
Fractures of the arm ............. 585
Fractures of the thigh ............ 602
France, duration of life in.......... 534
France, evils of the factory system in, 535
Freemantle, nosological report for.... 209
Functions of the spinal cord ........ 118
Functions of the sympathetic nerve.. 363
Fungoids of the lymphatic glands.... 141

G.

Ganglia of the sympathetic, office of the 366
Ganglionic enlargement of the pneumo-gastric nerve, Mr. Cock on the.... 361

Ganglionic enlargement of the pneumogastric nerve ................ 372
Gangrene, cases of, by Mr. Cooper ... 215
Gangrene, dry, of the hand.......... 215
Gangrene of the larynx ............ 396
Gastro-enteric fever ............... 475
Generation, physiology of ........ . 379
Germans, morals and manners of .... 537
Gizzard, anatomy of the............ 428
Gleaning in the Indian seas ........ 209
Gondret on derivation ............ 457
Grainger on the spinal cord.... 116, 361
Grant's outlines of comparat. anatomy 412
Granular affection of the kidneys .... 543
Grey & fibrous substances, properties of 121
Guerbois on aphorisms of Hippocrates 276
Gunshot-wound of the popliteal artery 283
Gunshot fractures, Mr. Guthrie on .. 585
Guthrie on injury of the shoulder.... 289
Guthrie's clinical lectures .......... 585
Guy's Hospital Reports........ 116, 213
H.
Hæmorrhage, fatal, from popliteal art. 514
Hæmorrhoidal affections .......... 297
Hæmorrhoidal vessels, congestion of 297
Hæmorrhoidal tumors, causes ...... 309
Hæmorrhoids, external, treatment of 323
Hand, dry gangrene of the ........ 215
Heart, Dr. Williams on palpitation of, 429
Heart, nervous palpitation of the ... 434
Heart, passive palpitation of the .... 435
Hepatic abscess bursting into the colon 573
Herpetic ulcerations from bubo .... 355
Hidrosis, case of.................. 579
Higher animals, digestive organs of the 424
Hilton on the laryngeal nerves ...... 361
Hilton on the sacculus in human larynx 361
Hippocrates, commentaries on the aphorisms of ................. 276
Hunter, John, works of ........ 65, 329
Hydrocele, injections of iodine in.... 563
Hydrocephalus acutus ............. 51
Hydrophobia, spontaneous.......... 555
I.
Iliac artery, spontaneous cure of an aneurysm of the ................ 567
Illustrations of phrenology.......... 225
Illustrations of acute phthisis........ 270
Imbibition, redness from............ 448
Imperforation of the anus .......... 4
Imperforation of the rectum ....... 4
Incision into the knee-joint ........ 513
Increase of insanity............ ... 533
Indian seas, gleanings in the ........ 209
Indolent syphilitic bubo............ 360
Inflamed tissues, fluid products of.... 449
Inflammation of the rectum ........ 18
Inflammation, nature of ........... 441
Inflammation, physical characters of, 444
Inflammation, redness & vascularity in 444
Inflammation, diagnostic characters of 447
Influence of climate on phthisis pulmon. 267
Influenza, Dr. Blakiston on the ..... 168
Inhabitants of Germany, morals and manners of ................... 537
Injections of iodine, in hydrocele .... 563
Injuries of the knee-joint.......... 512
Injury of the shoulder, Mr. Guthrie on 289
Insanity, remarks on .............. 531
Insanity, increase of .............. 533
Insanity, tables of ............... 534
Intense cephalalgia, case of ........ 553
Intermittent fever ................ 496
Intermittent fever, causes of........ 497
Intermittent fever, treatment of...... 498
Internal tumors .................. 313
Internal tumors .................. 301
Involuntary motions .............. 369
Iodine, injections of, in hydrocele.... 563
Ireland, bye-laws of the Royal College of Surgeons in................... 622
J.
Jamaica, flora of.................. 170
Johnson, Dr. and Dr. Dickson ...... 285
Johnson, Dr. on abdominal pulsation, 569
Johnson, Dr. his case of obscure disease 572
Johnson, Dr. on tympanitis abdominalis 576
K.
Kent and Canterbury Hospital, report of the .................. 177, 473
Kidneys, action of diuretics on...... 111
Kidneys, disease of the ............ 542
Kidneys, case of granular affection of, 543
Knee-joint, injuries of the.......... 512
Knee-joint, compound dislocation of 512
Knee-joint, incision into the........ 513
Knee-joint, wound of the.......... 514
L.
Laceration of the rectum ........... 13
Large blisters, utility of, in white swell. 550
Laryngeal phthisis, MM. Trousseau and Belloc on................ 50, 393
Laryngeal nerves, Mr. Hilton on the.. 361
Laryngeal nerves, distribution and function of the ................ 375
Laryngeal nerve, superior .......... 375
Larynx, description of the pouch in the 376
Larynx & trachea, Mr. Porter on, 50, 393
Larynx & trachea, Mr. Ryland on, 50, 393
Larynx, calcareous degeneration of the 394
Larynx, gangrene of the .......... 396
Larynx and trachea, foreign bodies in, 402
Larynx and trachea, wounds of the .. 406
Lawrence on ruptures ............. 471
Lawrence on encysted tumors of eyelids 515
Leeds' petition to Parliament ...... 580
Leg, treatment of varicose veins of the 221
Lepra ......................... 524
Lesions of the arachnoid .......... 451
Lesions of the pericardium.......... 455
Lesions of the pleura ............. 456
Lesions of the uterus ............. 545
Lesions of the uterine lymphatics .... 545
Lesions of the uterine veins ........ 546

Lesions of the peritoneum ......... 546
Lesions of the digestive tubes ....... 547
Letters testimonial, examination for.. 623
Leukosis ........................ 195
Leuret on insanity ................ 531
Lever's case of hidrosis ............ 579
Life, duration of, in France ........ 534
Limerick Infirmary .............. 512
Liquor lyttæ .................... 624
Local congest. from depending position 447
Loss of the memory of words ...... 243
Louis on auscultation.............. 257
Lower extremities, Mr. Burke on fractures of the ..................... 171
Lymphatic glands, tumors of ...... 137
Lymphatic glands, non-malignant scirrhus of the ..................... 138
Lymphatic glands, malignant tumors of 139
Lymphatic glands, fungoides of the .. 141

M.

M'Cleod on rheumatism .......... 223
M'Divitt's Report of the Kent and Canterbury Hospital ............ 177
Macfadyen's flora of Jamaica........ 170
Macilwain on medicine and surgery .. 470
Magendie on auscultatory phenomena 521
Magendie on contagion ............ 522
Magendie on absorption............ 527
Malformations of the rectum and anus, 3
Malignant tumors of lymphatic glands 139
Marshall's gleanings in the Indian seas 209
Mayo's philosophy of living ........ 161
Medical science & ethics, Dr. Porter on 163
Medical Almanack for 1838 ........ 175
Medical history, &c., of British Legion 387
Medulla oblongata ................ 132
Membranous angina, occurrence of, in scarlatina.......................... 488
Mercury, administration of......... 67
Mercury, method of administration of, 74
Mercury, treat. of syphilitic bubo by 347
Microscope, use of the, in physiological enquiries ................... 529
Mitcherlich on the action of diuretic medicines....................... 107
Monat on diseased veins............ 574
Monomania, Dr. Blake on.......... 84
Montgomery on pregnancy ........ 90
Morals and manners of Germans .... 537
Morbid anat. of the serous membranes 450
Motor oculi....................... 126
Mucous fever ..................... 476
Mucous discharge ... ............ 323
Mucous glands, tumors of the ...... 149
Mucous glands of the vagina, tumors of 152
Mucous secretions, transparent...... 39
Mucous secretions, opaque ......... 40
Müller's elements of physiology.. 116, 469
Muscular action, theory of.......... 368

N.

Nature of inflammation ............ 441
Nature of rheumatism ............ 538
Negative virtues .................. 620
Negro, organization of the brain in the 249
Nervous palpitation of the heart .... 434
Nervous palpitation, diagnosis of.... 436
Nervous system, Dr. Verity on the .. 166
Nervous system, classification of the.. 371
Nervous system, divisions of the .... 371
Nervous system of the amphibia .... 412
Nervous system, Swan on the comparative anatomy of the.......... 413
Neuralgia of extremity of the rectum, 24
Neuralgia of the testicles .......... 305
New French phrenological journal .. 236
New treatment of strictures of urethra 566
Nitric acid, poisoning by .......... 551
Nonat on puerperal fever .......... 545
Norfolk Island.................... 212
Nosological report for Freemantle.... 209

O.

O'Brien's report of cases in the County of Clare Infirmary ............. 281
Obscure disease, case of, by Dr. Johnson 572
Occurrence of pericarditis in rheumatism ...................... 223
Œsophagus and trachea, stricture of the 571
Office of the ganglia of the sympathetic 366
Operations, phlebitis after.......... 547
Ophthalmia, remarks on............ 556
Organization of the brain in the negro 249
Organs of calculation, alleged affection, 243
Ourang-outang, brain of the........ 251

P.

Palpitation of heart, Dr. Williams on, 429

Paris on pharmacologia ............ 464

Parliament, Leeds petition to ...... 580

Parotid of the rattle-snake.......... 429

Passive palpitation of the heart...... 435

Patella, fracture of, by muscular effort, 551

Pathology of tubercles ............ 264

Pathology of puerperal fever........ 545

Peculiar affection of the fore-arm.... 549

Penis, inflammation of absorbents of, 330

Perforation of the vertebral column by an aneurysm of the aorta ........ 568

Pericarditis, occurrence of, in rheumatism ...................... 223

Pericardium, lesions of the ........ 455

Peritoneum, lesions of the.......... 546

Pharynx, tumor of the ............ 151

Pharynx, wound of the ............ 283

Philosophy of living, by Mr. Mayo .. 168

Phlebitis ........................ 574

Phlebitis after surgical operations.... 547

Phrenological journal.............. 238

Phrenology, illustrations of ........ 225

Phrenology, in connexion with physiognomy ...................... 228

Phrenology, application of, to education .......................... 238

Phrenology, opposed to fatalism .... 241

Phthisis laryngea ................ 62

Phthisis pulmonalis, M. Bricheteau on 264

Phthisis, antimonial treatment of.... 265
Phthisis pulmonalis, influence of climate on .................... 267
Phthisis, acute, illustrations of...... 270
Physical characters of inflammation.. 444
Physical signs of bronchitis ........ 41
Physiological enquiries, use of the microscope in .................. 529
Physiology, Müller's elements of..116, 469
Physiology of generation ......... 379
Physiology of the spinal cord........ 130
Piorry on encephalic irritation ...... 252
Plague, is it contagious? .......... 525
Pleura, lesions of the.............. 456
Pneumogastric nerve, Mr. Cock on ganglionic enlargement of the .... 361
Poisoning by sulphuric acid ........ 284
Poisoning by nitric acid........... 551
Polygastrica .................... 418
Polypiphera..................... 420
Popliteal aneurysm, 2nd operation .. 219
Popliteal artery, gun-shot wound of the 283
Popliteal artery, fatal hæmorrhage of 514
Poriphera ...................... 420
Porter on the larynx and trachea..50, 393
Porter or medical science and ethics.. 163
Potatoes, raw, in scurvy .......... 553
Pregnancy, Dr. Montgomery on.... 90
Pregnancy, effects of.............. 91
Pregnancy, practical considerations of 93
Pregnancy, signs and symptoms of.. 96
Pregnancy, presumptive signs of.... 97
Pregnancy, probable signs of........ 100
Pregnancy, unequivocal signs of.... 102
Primary bronchitis, acute and chronic, 38
Prolapsus of the mucous membrane.. 304
Prolapsus of the rectum............ 323
Prolapsus of the rectum, treatment of 325
Prolapsus ani ................... 324
Prolapsus uteri, radical cure of...... 562
Prolonged lactation, fever from..205, 473
Properties of the discharge from bubo 340
Proximate cause of rheumatism .... 538
Puerperal fever, pathology of........ 545
Pyrexia, Dr. Clutterbuck on........ 153
Pyrexia, treatment of ............ 158

Q.

Quain's elements of anatomy....116, 469
Quain's anatomical plates.......... 116

R.

Radical cure of prolapsus uteri...... 562
Râles .......................... 262
Rattle-snake, parotid of the ........ 429
Rectum and anus, Dr. Bushe on diseases of the................ 1, 297
Rectum, diseases of the............ 297
Rectum, Mr. Syme on diseases of the 1, 297
Rectum and anus, malformations of the 3
Rectum, imperforation of the ...... 4
Rectum, unnatural termination of the 4
Rectum, termination of other organs in 5
Rectum, absence of the............ 5
Rectum, foreign bodies in the...... 9
Rectum, prolapsus of the .......... 323
Rectum, laceration of the.......... 13
Rectum, inflammation of the........ 18
Rectum and anus, inflammation of the, from gonorrhœal matter ........ 20
Rectum, neuralgia of the extremity of 24
Rectum, ulceration of the .......... 31
Redness from imbibition .......... 448
Reflex function of the spinal cord.... 118
Relaxation of the anus ............ 328
Remarks on insanity .............. 531
Remarks on epidemic diseases...... 554
Remittent fever of children ........ 481
Remittent fever of do., treatment of.. 482
Report from Dumfries & Galloway infir. 205
Report from Kent & Canterbury Hosp. 473
Report of Western Lying-in Hospital 507
Resonance of voice, modifications of 261
Respiratory murmur, absence of the 259
Rheumatism, tables relating to...... 223
Rheumatism, duration of .......... 223
Rheumatism, liability of diff. ages to 223
Rheumatism, nature of ............ 538
Rheumatism, utility of camphor in .. 554
Ruptures Mr. Lawrence on ........ 471
Ryland on the larynx and trachea..50, 393

S.

Sacculus in the human larynx, Mr. Hilton on the.................. 361
St. George's hospital .............. 220
Saline diuretics ................. 111
Salivary glands, tumors of the ...... 148
Sanson on amaurosis and cataract.... 559
Scirrhus of the lymphatic glands .... 138
Scirrhus ....................... 145
Scrofulous glandular tumors........ 137
Scrotum and thigh, sinus between the, 359
Scurvy, raw potatoes in............ 553
Secondary bronchitis, treatment of .. 49
Secreting glands, tumors of the...... 142
Sensation and volition, seat of ...... 119
Serous secretions ................ 40
Serous membranes, morbid anatomy of 450
Shoulder, Mr. Guthrie on injury of.. 289
Signs of pregnancy................ 96
Simple continued fever ............ 190
Sinus between the scrotum and thigh, 359
Situation of syphilitic buboes........ 334
Spasmodic contraction of sphincter ani 26
Speculum in diseases of the uterus .. 519
Spermatic animalculæ, experiments on 329
Sphincter ani, spasmodic contraction of 26
Sphincter ani, spasmodic contraction of the, with affection of the genito-urinary organs ................ 29
Spinal cord, Mr. Grainger on the.... 116
Spinal cord, structure of the........ 118
Spinal cord, reflex function of the .. 118
Spinal cord, anatomy of the ........ 123
Spinal nerves, anatomy of the...... 124
Spinal cord, physiology of the ...... 130

Spinal cord, vertebral portion of the.. 131
Spinal cord, Mr. Grainger on the.... 361
Spinal cord, anatomy of the........ 361
Spinal arachnoid.................. 454
Spontaneous hydrophobia........... 555
Stearine, composition of .......... 459
Sterility, causes of, in women ...... 529
Stimulating diuretics ............... 110
Stokes on diseases of the chest...... 33
Strictures of the urethra, treatment of 566
Stricture of the œsophagus & trachea 571
Strumous ophthalmia, treatment of.. 516
Sub-maxillary ganglion, anatomy of 366
Sulphuric acid, poisoning by........ 284
Suppression of the menses as a sign of pregnancy .................... 97
Suppuration, treatment of bubo in .. 347
Surgical aphorisms................. 556
Surgical pathology of the larynx and trachea. By W. H. Porter, Esq... 50
Swan on the comparative anatomy of the nervous system............... 412
Syme on diseases of the rectum...... 1
Syme on diseases of the rectum. .... 297
Sympathetic nerve, functions of the.. 365
Symptomatic pyrexia............... 154
Synovial rheumatism....... ...... 224
Syphilitic bubo, indolent ........... 360
Syphilitic bubo .................... 333
Syphilitic bubo, treatment of........ 347
Syphilitic buboes, situation of ...... 334

T.

Tedious labor, cases of............ 509
Tendo-achillis, division of the ...... 221
Theobroma ....................... 171
Theory of muscular action....... .... 388
Thigh, fractures of the ............ 602
Thigh-bone, excision of the head of the 608
Thoracic disease, diagnosis of ...... 34
Ticknor's philosophy of living ....., 161
Tongue, tumors of the ............ 150
Tongue, fatal hæmorr. from wound of 217
Treatment of bronchitis............ 47
Treatment of bubo................. 341
Treatment of venous hæmorrhoids .. 321
Triturating apparatus of animals .... 426
Trosseau & Belloc on laryneal phthisis 50, 393
Tubercles, pathology of............ 264
Tubercles, composition of... ....... 265
Tumor of the pharynx ............ 151
Tumors, Dr. Warren on . ........ 136
Tumors of lymphatic glands........ 137
Tumors of the secreting glands...... 142
Tumors of the breast.............. 142
Tumors of the salivary glands ...... 148
Tumors of the mucous glands ...... 149
Tumors of the tongue..... ......... 150
Tumors at the verge of the anus .... 307
Turner's elements of chemistry...... 176
Twining, the late Mr............... 581
Tympanitis abdominalis .......... 576
Typhoid fever..................... 191
Typhus, bronchitis of...... ....... 44
Typhus, contagiousness of ........ 523

U.

Ulceration of the rectum .......... 31
Unequivocal signs of pregnancy .... 102
Ununited fracture, case of.......... 216
Urethra, new treatment of stricture of 566
Urine, constituents of ............ 108
Urine, case of retention of the ...... 281
Use of the microscope in physiological enquiries ....................... 529
Uterine lymphatics, lesions of the.... 545
Uterine veins, lesions of the........ 546
Uterus, lesions of the ............ 545
Uterus, extirpation of the.......... 561
Uterus, speculum in diseases of the .. 519
Utility of large blisters in white swellings ........................ 550

V.

Vapour, Dr. Wilson on the curative effects of.................. ... 467
Varicose veins of the leg, treatment of 221
Velpeau on xerophthalmia.......... 558
Velpeau on the treatment of burns.. 562
Venereal disease, Dr. Colles on the, 65, 329
Venereal disease, Dr. Wallace on the 329
Venous hæmorrhoids............... 307
Venous hæmorrhoids, treatment of.. 321
Veratria, employment of .......... 555
Verity on the nervous system....... 167
Version cases ..................... 510
Vertebral column, perforation of the, by an aneurysm ................. 568
Villeneuve on epidemics............ 554
Vito Mangiamele, account of........ 247
Voice, resonance of the............ 258
Voice, modifications of resonance of.. 261
Voluntary motion, theory of........ 368

W.

Walker on the eye................ 471
Wallace on the venereal disease .... 329
Walther's surgical aphorisms ...... 556
Warren on tumors................. 136
Western Australia, state of health in 210
Western Lying-in Hospital, report of 507
Westminster Hospital ............ 289
White swellings, utility of large blisters in............................ 550
Williams on palpitation of the heart 429
Wilson on vapour applied locally.... 467
Windpipe, diseases of the .......... 393
Women, causes of sterility in ...... 529
Words, loss of the memory of ...... 243
Works of John Hunter..........65, 329
Wound of the tongue, fatal hæmorrhage 217
Wound of the pharynx ........... 283
Wound of the knee-joint .......... 514
Wounds of the larynx and trachea .. 406

X.

Xerophthalmia, case of ............ 558

Y.

Yellow fever ..................... 524

